# Micro-mechanical and tribological behavior of Al/SiC/B_4_C/CNT hybrid nanocomposite

**DOI:** 10.1038/s41598-023-39713-2

**Published:** 2023-08-12

**Authors:** A. Nirala, S. Soren, Navneet Kumar, Mohammad Amir Khan, Saiful Islam, Nadeem A Khan

**Affiliations:** 1 Department of Fuel, Minerals and Metallurgical Engineering, Indian Institute of Technology (ISM) Dhanbad, Dhanbad, India; 2 Department of Mechanical Engineering, Galgotias College of Engineering and Technology, Greater Noida, India; 3 Department of Mechanical Engineering, Maharana Pratap Polytechnic, Gorakhpur, Uttar Pradesh 273015 India; 4 Department of Civil Engineering, Galgotias College of Engineering and Technology, Knowledge Park I, Greater Noida, Uttar Pradesh 201310 India; 5 Civil Engineering Department, College of Engineering, King Khalid University, Abha, Asir 61421 Kingdom of Saudi Arabia; 6 Interdisciplinary Research Center for Membranes and Water Security (IRC-MWS), King Fahd University of Petroleum and Minerals, Dhahran 31261, Saudi Arabia

**Keywords:** Civil engineering, Mechanical engineering

## Abstract

The aluminum nanocomposite is fabricated through squeeze stir casting method where CNT, SiC/B_4_C powder has been used as a reinforcement in an aluminum matrix. Squeeze action in stir casting opted due to proper reinforcement of 2 vol% of CNT in the matrix. The boron carbide and silicon carbide have been added by 8 and 12 vol% in the matrix. Uniform distribution of reinforcement and phase analysis has been shown by scanning electron microscopy (SEM) and XRD analysis. The formation of intermetallic compounds like Al_3_BC and Al_4_C_3_, dislocation forests, and the interaction of the reinforcement with the matrix are all confirmed by transmission electron microscopy (TEM). The micro-mechanical behavior of aluminum nanocomposites was investigated using nano indentation. The nano hardness, Vickers hardness, and Young's modulus of 12 vol% B_4_C compared with 12 vol% of SiC are increased by 12%, 23%, and 16%, respectively, and the same trend has been observed for the 8 vol% B_4_C reinforced composite. The model analysis for Young's modulus has been done and the experimental value for the modulus of elasticity of the composite are validated and not find such differences significantly. The surface topography was determined, furrow scratches and wear scars, and it was discovered that B_4_C reinforced composites have reduced stripping pits inside the wear marks, as well as lower wear width and depth. Wear analysis is essential because abrasive encounters result in substantial damage owing to larger pits and bigger wear scars.

## Introduction

Aluminum hybrid composites (AHCs) are widely employed in a variety of industries, including automotive, marine, military, aerospace, constructions, and transportation, where high material strength is required to withstand induced stresses^1–4^. AHCs are widely used due to their superior mechanical qualities. In a previous study, it was also demonstrated that the intrinsic ductility of aluminum/aluminum alloys declines when reinforcement is added such as SiC, Al_2_O_3_, TiB_2_, or graphite^5,6^. Throughout the years, SiC^7^ as reinforcement has drawn a lot of attention from researchers, and it also has outstanding physical properties and is one of the easiest substances to form chemical bonds with an aluminum matrix^8^, whereas B_4_C has low density, high stiffness, and superior hardness behavior^9,10^. Various studies have already evaluated the mechanical and physical properties of composite materials, and it is enhanced by ceramic particles made along with various aluminum alloys matrix. Ravi Kumar et al.^11^ studied the mechanical properties of Al/TiC composites, and their findings show that the addition of TiC reduces the composites' density, impact strength, and elongation. Aluminum matrix composites were produced using varied weight % of 6, 8, 10, and 12, B_4_C particulates to explore composite characteristics, and It was found that the tensile strength and hardness continuously increased when the particles were added to the matrix^10,12^. Ghanbari et al.^13^ investigated composites made with aluminum matrix along with SiC particles, finding that the hardness of the composites rises as a result of heat treatment, as well as the creation of tiny grains in the microstructure. The influence of SiC particulates on tensile behavior, hardness, and density in composites was evaluated in their study, and they have discovered that tensile strength, hardness, and porosity in composites increased to a substantial level, but composites' density and impact strength reduced^14^. Some of the authors have fabricated an aluminum composite with SiC (0, 5, 10, 15, and 20 wt%) and found that maximal reinforcement at 20 wt% SiC results in the highest hardness and tensile strength. Comparatively 5 wt% reinforced composite has a higher hardness and tensile strength than 10 wt% SiC reinforced composite^15^. The authors have also investigated the mechanical and machining characteristics of aluminum metal matrix composites made with MoS_2_ and B_4_C particles, and also conducted tests to investigate surface roughness and forces for deformation during the turning operation of the specimen^16,17^. Afkham et al.^18^ conducted a comprehensive analysis on aluminum nano composite where aluminum used as matrix and Al_2_O_3_ nanoparticles as reinforcement, and found that the fabricated composites had increased tensile strength and hardness. Senel et al.^19^ studied the hardness and compressive strength of graphene nanoplatelets (GNPs) and SiC-reinforced aluminum composites. They found that hardness and compressive strength improved by increasing the amount of reinforcement such that SiC and GNPs. Singh and Goyal^20^ have been studied the effect of reinforcement of SiC and B_4_C in aluminum alloy (AA6082) matrix and concluded as hardness increases by increasing the reinforcement while reduces the wear rate of the composite. Despite the improved features of aluminum nano composite, the current industrial manufacturing processes are neither reliable nor cost-effective for the manufacture of bulk composites. The most widely utilized methods for producing aluminum nano composite are friction stir processing, liquid state (conventional^21^, squeezing^7^, and ultrasonic cavitation^22^) stir casting, and powder metallurgy^23^. The achievement of homogeneous dispersion of nano-sized particles is the primary challenging problem for the production of aluminum nano composite, and it is due to poor wettability between the reinforcement and matrix materials. The poor wettability was caused by the significant disparity in densities between nano-sized particles and the matrix alloy, as well as the significantly greater specific surface area of the reinforcement nano particles^24^. As a result, some authors have employed ultrasonic stir casting and powder metallurgy to create evenly dispersed composites, however these methods are slightly more expensive and inefficient than squeeze stir casting^25^. Squeeze stir casting has been employed to resolve the problem and spur industrial viability.

According to the previous literature, there are a rare study on (SiC + CNT) and (B_4_C + CNT) reinforced composites using aluminum matrixes. As a result, there is a significant opportunity to motivate the fabrication of nano composites and the comparison of mechanical, physical, and wear characteristics. The lightweight structures, parts, and components made from Al/SiC/B_4_C/CNT hybrid nanocomposite may be utilized to build aeroplanes, satellites, spacecraft, and vehicles. It is the perfect material for various applications due to its excellent strength to weight ratio. The reason a tribology study required to conducted, because the current research is aimed at the aforementioned application. The study of tribological properties is required for aluminum composite materials to understand the wear and friction behavior of the material, which can help optimize the design and performance of the material. To ascertain how the materials will function in practical applications, this may include putting them through a series of tests at various pressures, temperatures, and speeds. Studies on wear morphology entail looking at a material's surface after it has experienced wear. Because it may shed light on the material's durability and wear resistance, this kind of research is crucial for aluminum composite, which is utilized in automotive applications.

In view of this, the authors wish to emphasize and characterize the aluminum nano composites and make an effort to advance knowledge in this field by comparing the mechanical, physical, and wear behavior of the (SiC + CNT)/Al and (B_4_C + CNT)/Al composites, which covers research into yield strength, friction, and wear behavior as well as density, porosity, microhardness, and nanohardness. Pure aluminum is employed as a matrix material in the present study to determine the best alternative to aluminum alloy^26^. The authors have attempted to individually construct (B_4_C + CNT)/Al and (SiC + CNT)/Al composites using squeeze stir casting with varied volume % of 0, 8, and 12. Additionally, optical micrographs, SEM micrographs, TEM images, and XRD patterns were used to study the phases and microstructure of the composites. The purpose of this work is to compare the characteristics of the composites utilizing the same basic matrix and with the identical testing procedures.

## Materials and methodology

### Reinforced particulates

**Figure 1:**
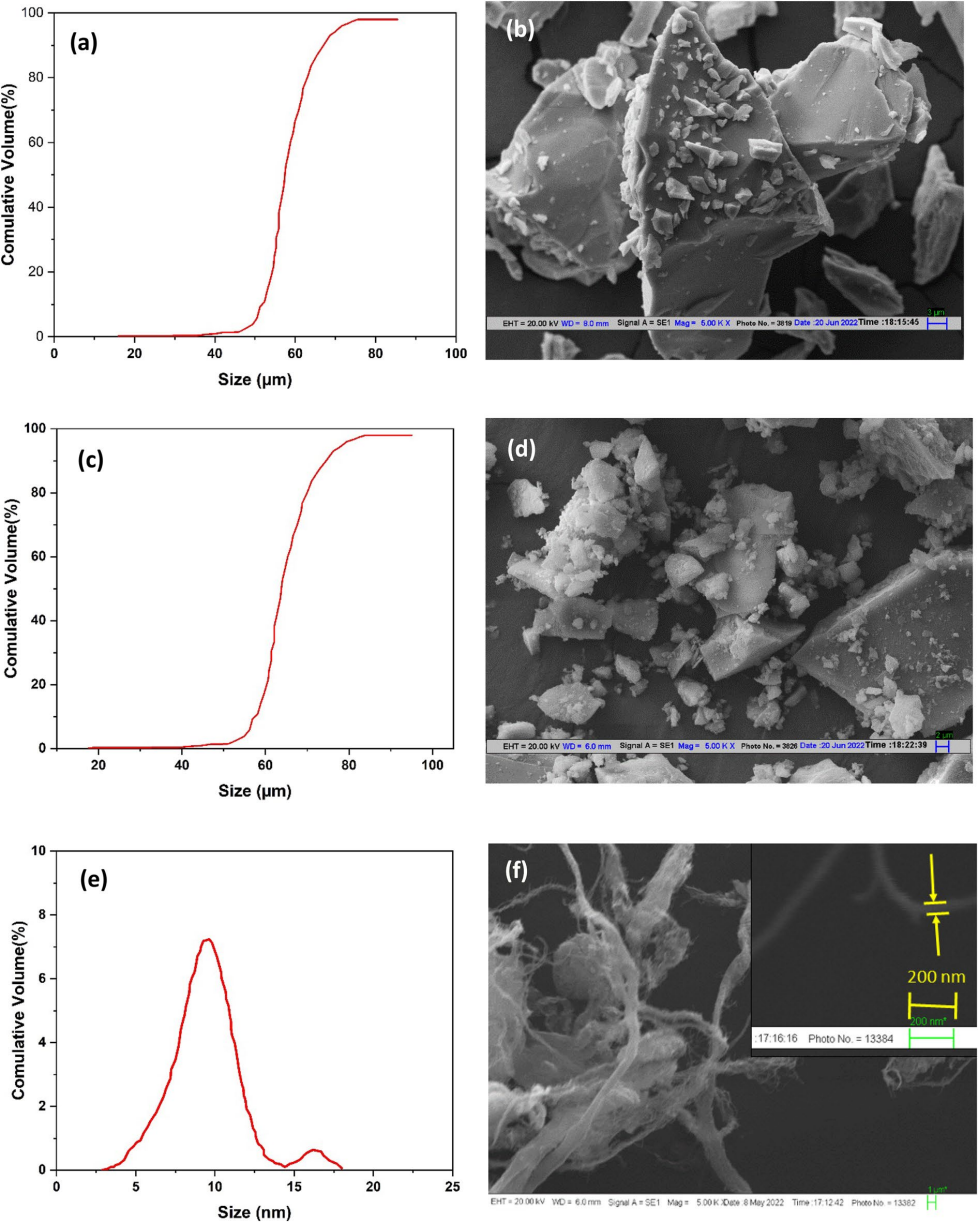
Particulate morphology (**b**) B_4_C (**d**) SiC and (**f**) MWCNT and particle size distribution diagram of (**a**) B_4_C (**c**) SiC and (**e**) MWCNT.

A unique and efficient method for producing unconventional aluminum matrix composites is hybrid reinforcing. It is envisaged that by combining two or more reinforcements, their synergistic effects would be investigated, the limits of a single reinforcement would be overcome^27–30^. In the present study to make the composites, pure aluminum (99.9% pure) was used as the matrix metal, and fine particulates of B_4_C, SiC, and CNT were added to the matrix. Particulate reinforcement B_4_C and SiC were obtained from Parshwamani Metals in Mumbai, India, while MWCNT (Multi-wall carbon nanotube) was provided by ad-nano pvt ltd, Chennai, India. The particulates details have shown in Tables 1 and 2, while Fig. 1 shows their SEM images of powders with their average particle size diagram. Boron and silicon carbide is in irregular shape and almost same in size. The composite's composition is prepared according to Table 3 and fabricated using the squeeze stir casting method. For a better mechanical outcome, the reinforcement composition has been chosen based on prior literature^26,31^. CNT was mixed of 2 vol% to the matrix materials because it demonstrated improved wear and mechanical characteristics^4^.

**Table 1:** Details of the B_4_C and SiC particulate materials.

S. no	Reinforced particulates	Average particle size (µm)	Density (g/cm^3^ )	Melting point (°C)	Hardness (H_V_)
1	SiC	56	3.18	2700	285^32^
2	B_4_C	52	2.51	2450	305^32^

**Table 2:** Multi-wall carbon nanotube (MWCNT) specification.

Behavior	Details
Type	MWCNT
Color	Black powder
Purity	> 99%
Average diameter	10–15 nm
Average length	~ 5 μm
Amorphous carbon	< 1%
Surface area	~ 400 m^2^/g

**Table 3:** Nomenclature of composite for easy interpretations.

S. no	Composition (in vol%)	Nomenclature
1	Al (90) + B4C (8) + CNT (2)	ABC
2	Al (86) + B4C (12) + CNT (2)	ABBC
3 4	Al (90) + SiC (8) + CNT (2) Al (86) + SiC (12) + CNT (2)	ASC ASSC

### Powder morphological study

The particle size distributions of boron carbide, silicon carbide, and carbon nanotube are shown in Fig. 1a,c,e, respectively, and shown that particulates B_4_C and SiC have no significant differences in size. The sizes of the powders range from 1 to 80 µm for B_4_C and SiC powder, and there are no significant differences in average size between the powders. Both powder morphologies exhibit an irregular edge (shows in Fig. 1b,d,f), which effectively works for fabricated composite to prevent grain boundary sliding during fracture. Round edge powder has a lower mechanical withstand than rough surface powder, because rough surface hinders the grain boundary sliding at the time of fracture^33,34^.

### Method of fabrication

**Figure 2:**
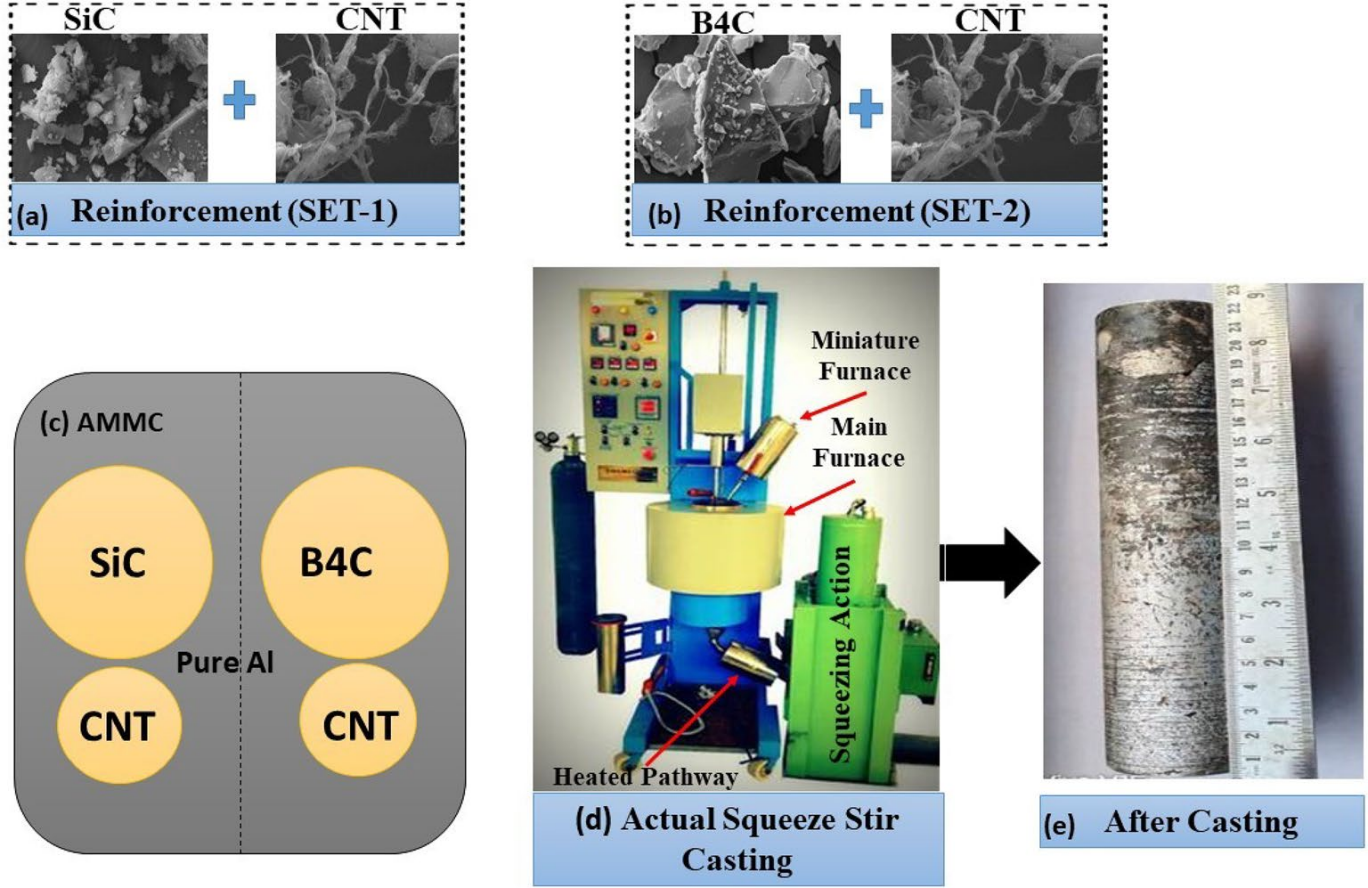
Reinforcement combination of the composite (**a**) SiC + CNT (**b**) B_4_C + CNT (**c**) schematic diagram of AMMC (**d**) actual setup of squeeze stir casting and (**e**) cylindrical slab (D = 50 mm, L = 220 mm) after casting.

Squeeze casting combines casting and forging techniques and is done while casting is under high pressure, making it one of the better fabrication methods compared to conventional stir casting method. The complete process of fabrication through squeeze-stir casting process is shown in Fig. 2, which is employed in this investigation and it is a bottom pouring type casting. Where, Fig. 2a–e shows (SiC + CNT), (B_4_C + CNT), schematic diagram of AMMC, actual setup of squeeze stir casting and fabricated cylindrical slab respectively. The current experimental setup has two furnaces, on the same platform, both of which are mounted. One is a main heating furnace, while the other is a miniature furnace. A PID controller controls both of them for on/off operation. The aluminum ingots (about 1.1 kg) are melted in the main heating furnace and mixed with two different additives [potassium fluorotitanate (K_2_TiF_6_) and 1 vol% magnesium] at a temperature of 800 ± 50 °C. These additives are used to enhance wetting capacity of the reinforcement in aluminum matrix^26^. And also, the reinforcements (B_4_C, SiC) were heated in the miniature furnace (at a temperature of 200 ± 25 °C) for 60 min to boost their wetting ability and lower their moisture content, then poured in to the molten matrix. After pouring the (SiC/B_4_C) reinforcement, the CNT was heated in a miniature furnace to 200 ± 25 °C using the same process. To lower the bath temperature, the furnace is turned off, which causes vortex formation to begin. Motorized up and down movement of twisted steel blades created the vortex in the furnace and then mixed preheated reinforced in the melt. The increased surface area of nanoparticles causes agglomeration or clustering in the molten melt, and this happen very rarely due to proper wetting behavior of particulates to matrix. Some authors have manufactured nanocomposites by incorporating Al_2_O_3_ nanoparticles into a semi-solid Al–Cu-alloy matrix with a lower Al_2_O_3_ content^35^. A heated tube passage carried the melt from the primary furnace to the permanent mold (kept at 650 °C to avoid pathway solidification) where squeeze casting took place for uniformly mixed melt. The permanent mold is made up of a hydraulic mounting piston (pressure of 150 MPa, piston diameter of 50 mm) that was used to draw the melt and keep it at that pressure until it cooled to room temperature. The tube between the heated pathway and the permanent mold has a smaller cross-sectional area. Lower cross-sectional area resulting in more surface area for greater heat dissipation and quicker solidification than mold. The molten metal was kept at a high pressure, the piston was raised to the necessary level, solidification began more quickly in the lower cross section region, and the mold was locked, confirming that there was no flow back into the furnace. The fabricated solid composite was removed from the mold and made ready for further specimen preparation. The same and separate casting methodology was used for each composition, as shown in Table 3.

### Microstructural examination and phase analysis

Circular cross-section samples were machined using wire EDM for the microstructural investigation. The multiple grades (400, 600, 800, and 1000) of abrasive paper were utilized for polishing the samples. Alumina nano powder suspended water slurry is used for cloth polishing on a polishing wheel at constant RPM to achieve shiny surface. The cloth polishing is completed until the surface is free of scratches and completely shiny surface. The samples must be thoroughly etched to disclose the micro-grain and for that Keller's reagent solution was employed in this study, which contained HCl (3 ml), HF (2 ml), H_2_O (175 ml), and HNO_3_(20 ml). Using a digital image acquisition optical microscope (model: DM2500, supplier: Leica Imaging System Ltd., Cambridge, UK), were used to captured optical microstructures. The images were captured at magnifications of 200× and 500×. The elemental distribution/mapping was studied using energy dispersive X-ray spectroscopy (EDS) analysis and the scanning electron microstructures of the specimen were captured using a scanning electron microscope (SEM, ZEISS EVO 50 operated at 5–20 kV) (OXFORD Instrument, INCA Penta FETx3). Aluminum composite specimens do not need a gold coating because they are naturally nonmagnetic.

Transmission electron microscopy (TEM), carried out at 200 kV, is used to describe the phases and dislocations present in the fabricated composite specimens. Thin slices (900 μm) are cut for the TEM sample preparation, which is followed by mechanical grinding to a thickness of 100 μm. Additionally, a Gatan disc punch was used to create a 3 mm diameter disc (Model number: 1104). Afterward, the Gatan precision ion polishing equipment was used to perform ion milling to create a transparent area (Model 691).

X-ray diffraction can distinguish between the crystalline site and phase present in the composite. The structure of a composite material may be examined using X-ray diffraction by measuring the angles and intensities of the diffracted X-rays. This makes it possible to ascertain the material's composition, structure, and qualities. X-ray diffraction can be used to investigate phase or any intermetallic formations that occur during casting. The polished and clean specimen was put in a holder and utilized to analyses the phases involved in the development of any intermetallic during fabrication. The computer was used to record the diffraction data of the specimen. Cu Ka (λ = 1.54 Å) was used to obtain XRD data using a Bruker D8 FOCUS X-ray with a scan rate of 1°/min between 20° and 90°.

### Mechanical characterization

The prepared composite specimen was tested for micro-hardness (Vickers hardness) and nano-indentation as part of the mechanical characterization. The micro-hardness measured using Vickers micro hardness tester (Bareiss VTP 6046 Bj07) at 0.1 kg load (with dwell time of 10 s). The indentation was located using a diamond indenter, and the hardness of the indentation was tested after that. The diagonal length is measured by live microstructure of composite by computer assisted optical microscope. This was accomplished using the ASTM E-384 test procedure. The hardness value has been averaged after being measured ten times. Using Eq. (1), the hardness value is calculated:

1 $${\text{H}}_{{\text{V}}} = { 1}.{\text{845F}}/{\text{d}}^{{2}}$$

where F is applied load and average diagonal length of the indented shape is d (d = (d_1_ + d_2_)/2).

**Figure 3:**
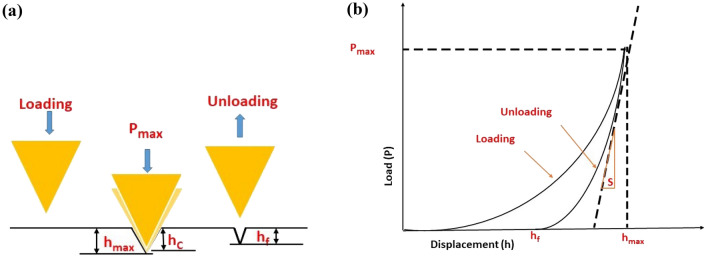
Schematic diagram (**a**) during nano indentation, the contact between the indenter's tip and the surface of the specimen and (**b**) typical nano indentation load–displacement curve.

Nano indentation tests were performed on the electro-polished nanocomposite prepared specimen. Figure 3a,b shows a schematic diagram of the indenter tip making contact with the specimen and then rebounding up to measure the strain or displacement in the composite after it has been unloaded. In the scanning probe microscope, the diamond tip was employed with a maximum peak load of 70 mN, a triangular loading–unloading function was used. Without any holding period, the peak load was reached in 5 s and removed again in 5 s. Each specimen had more than 30 indentations done at random areas in order to get accurate findings. For each composite specimen, the load vs displacement curves and the hardness and Young's modulus were assessed. The diagram displays the maximum applied load (P_max_), the maximum penetration depth (h_max_), the contact depth (h_c_), the final depth (h_f_), and the maximum strength (unloading Stiffness-S).

### Tribological testing

**Figure 4:**
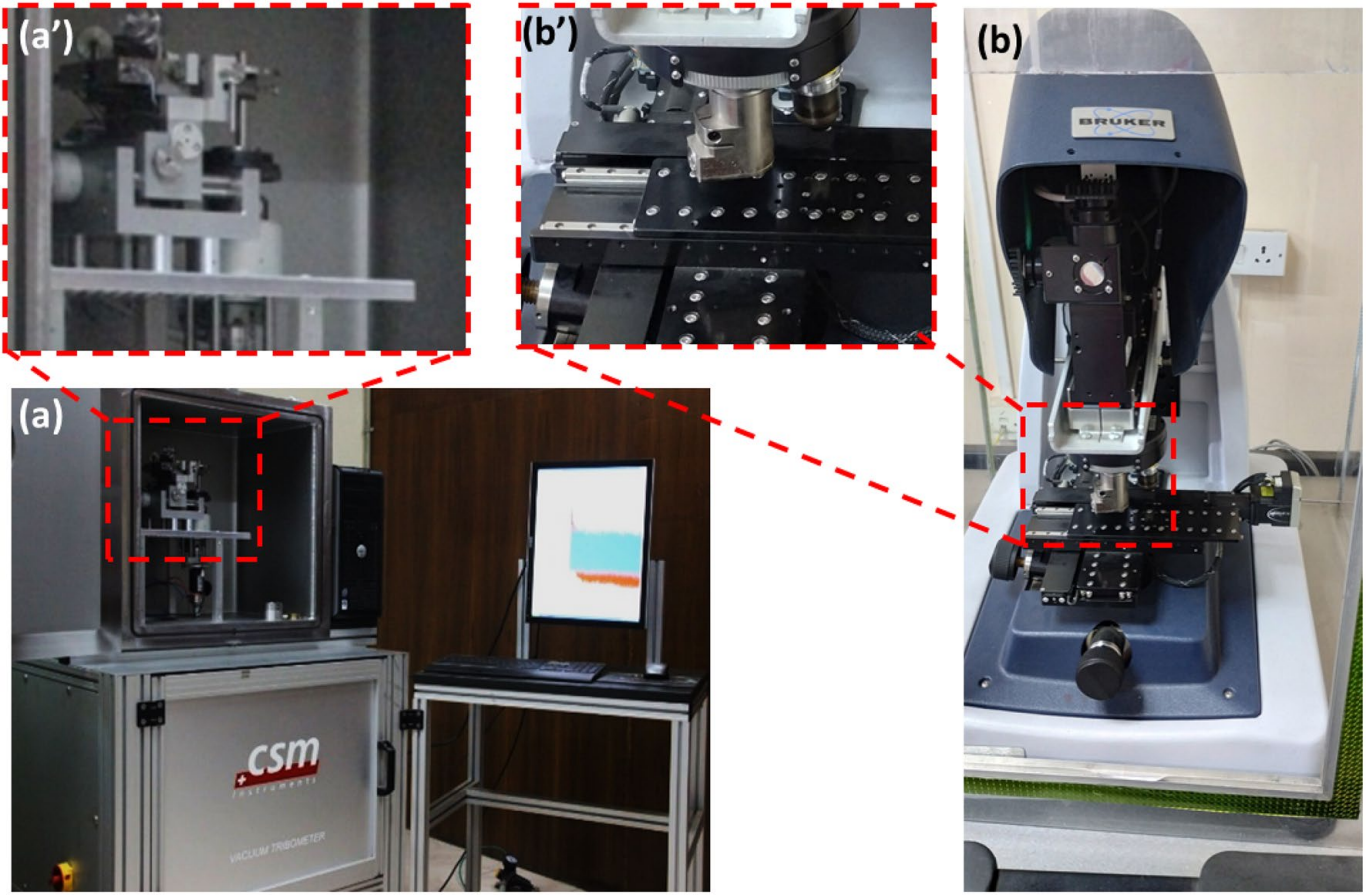
Machine used (**a**) wear/friction tester for ball on disc type (**a′**) Specimen holder for wear/friction test (**b**) 3D-optical surface profilometry (**b′**) Surface profile imaging stage.

The friction tests were conducted using the CSM ball on disc type wear tester (CSM Instrument, Switzerland), which is shown in Fig. 4a, and Fig. 4a′ is slot for specimen holder. Using linear reciprocating (stroke length 5.5 mm for the duration of 800 s and sliding distance of 880 cm) friction, we allowed a stainless steel ball (1Cr18Ni9Ti) with a 6 mm diameter to move over the surface of an aluminum composite specimen (ASTM G-133) under conditions of ambient temperature between 25 and 40 °C and relative humidity between 15 and 35%. Utilizing a high vacuum control kit, the tribometer chamber was used to measure the depth of penetration, coefficient of friction, and friction force in the time domain. The data were then displayed and explained using a computer interface. Figure 4b,b′ is 3D-optical surface prifilometery (OSP) machine and stages for the imaging, respectively.

The three-dimensional surface profiler MicroXAM-800 (Bruker) was utilized to assess the roughness of the surface and volume loss of the composite, and the friction tester's sensor recorded the friction coefficients in real time. The wear volume data was plugged into a formula (Eq. 2) to get the wear rates. When a ball exerts a normal load of 5 N on a specimen's surface, a wear scar or scratch surface is produced. Cross-section of the wear groove multiplied by its length gives volume of the wear scar. The volume calculation that made contact with the ball has been completed and specific wear calculated using Eq. (2):

2 $${\text{w}} = {\text{ dV}}/({\text{dL}} \times {\text{dF}})$$

where w is the specific wear rate, which corresponds to the composite's wear volume at unit applied load (dF), and unit slipping space (dL), V is the volume loss (mm^3^), L is the sliding distance (m), and F is the applied load (N), For the purpose of obtaining the average values, all tribological tests were conducted five times under identical experimental parameter. Since, wear or abrasion behavior of the materials involved, the nano thickness of the composite's outer layer must be studied concurrently. The selection of essential mechanical characteristics for nanometer thickness is required that’s why the nano indentation testing method is used in the current investigation.

The measurement of a material's surface topography or surface roughness is done using a technique called surface profilometry. It is a non-contact, non-destructive technique that can assess parameters including roughness, waviness, and flatness as well as surface characteristics like peaks, valleys, and grooves. In order to measure a material's surface using surface profilometry, a stylus or probe is commonly used. Height measurements are taken at certain locations along the surface as the stylus travels over it. A three-dimensional representation of the surface is then created using these height measurements, from which a variety of surface topography data may be extracted.

## Experimental results and discussion

### Phase formation and their analysis

**Figure 5:**
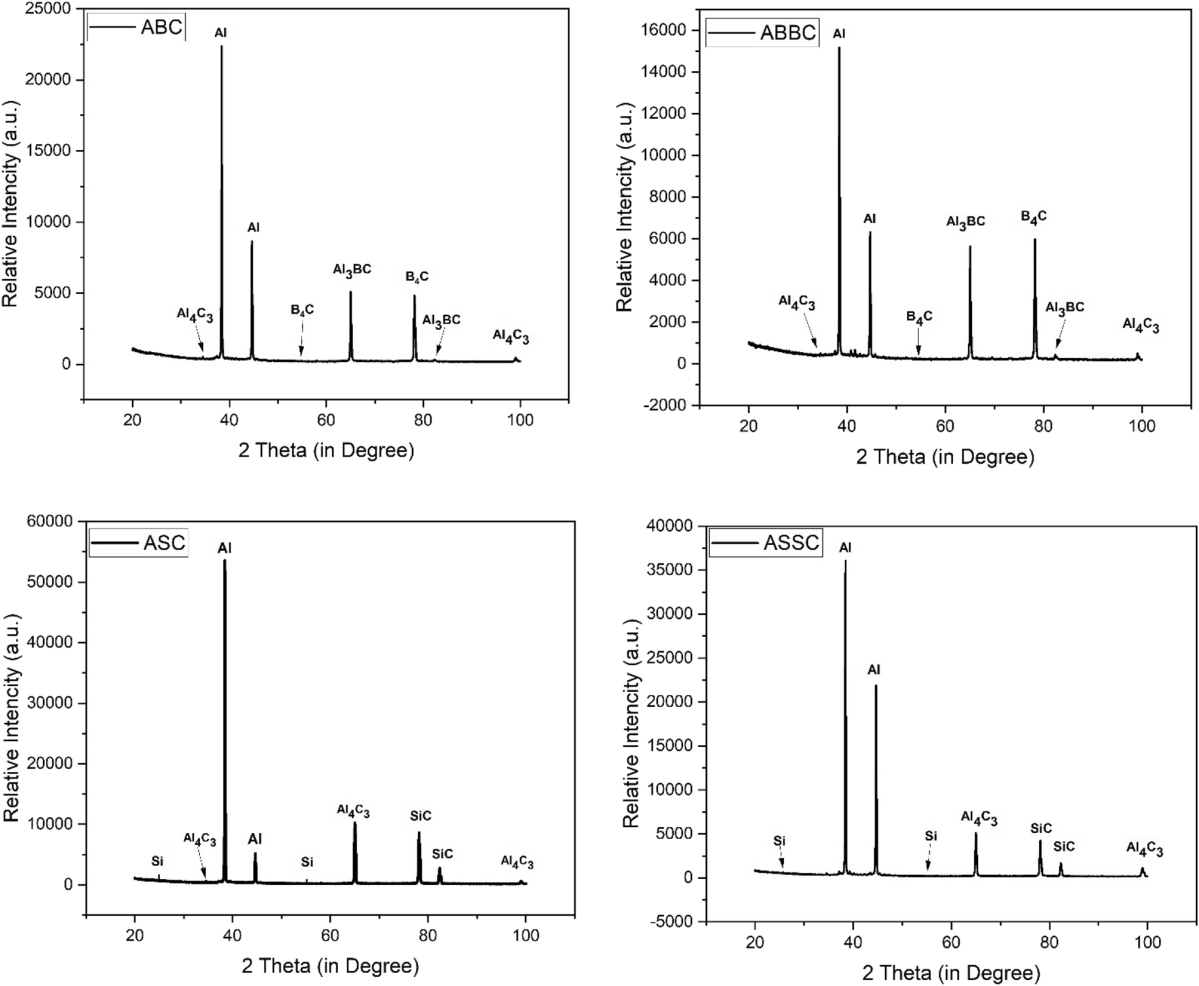
XRD phase patterns of ABC, ABBC, ASC, and ASSC composite.

The X-ray diffraction patterns of ABC, ABBC, ASC, and ASSC composite are shown in Fig. 5. B_4_C was reinforced with 8 and 12 vol% in the aluminum matrix and formed ABC and ABBC composites. Peaks of B_4_C, Al_3_BC, Al_4_C_3_, and Al are visible in the XRD patterns of ABC and ABBC. The XRD patterns peak of Al_3_BC are seen in both composites (ABC and ABBC) and it is due to the reaction between B_4_C and aluminum. The peak height of Al_3_BC has the small in ABC but comparatively longer in ABBC which indicates higher reinforcement of B_4_C in aluminum matrix. Similarly, the reinforcement of SiC mixed in aluminum matrix with 8 and 12 vol% and formed ASC and ASSC composites, respectively. At different diffraction angles, the XRD patterns in ASC and ASSC show different phase peaks such as Al_4_C_3_, SiC, Si, and Al. All available peaks indicate the presence of various phases in the composite. According to XRD patterns, every composite contains Al_4_C_3_ (brittle intermetallic) phase, which is not desired in a composite. It is formed by the reaction of aluminum hydroxide (aluminum reacts with moisture and forms aluminum hydroxide) with carbon (from carbide and CNT) to form Al_4_C_3_. The formation of Al_4_C_3_ can be reduced by optimizing process parameters such as coated reinforcement or matrix modification^36,37^.

### Microstructural analysis

The most important metallurgical characterization technique is optical microstructure because it is a key component of the visual inspection approach for the metal/alloys/metal matrix composite. SEM is utilized to visualize the reinforcement and matrix interface morphology of the composite at a greater magnification.

**Figure 6:**
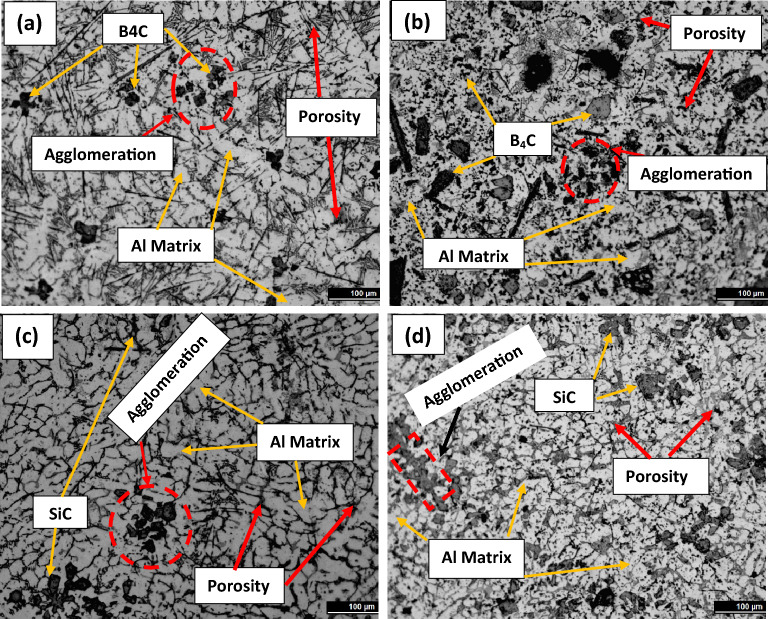
Optical microstructures of (**a**) ABC, (**b**) ABBC, (**c**) ASC, and (**d**) ASSC composites.

**Figure 7:**
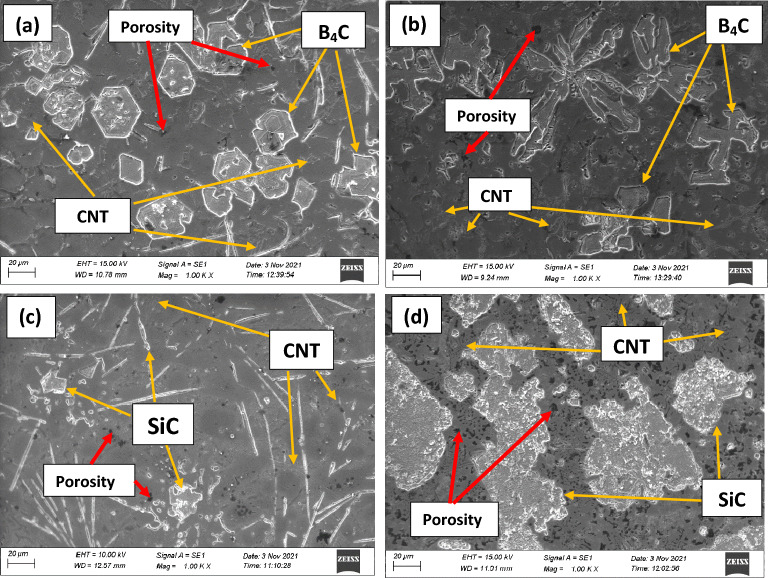
SEM microstructures of (**a**) ABC, (**b**) ABBC, (**c**) ASC, and (**d**) ASSC composites.

Figure 6 shows optical micrographs of composites with varied reinforced (boron carbide, silicon carbide, and CNT) vol% of 8 and 12, demonstrating that the mixture obtained was considerably homogenous. Similarly, Fig. 7 shows SEM micrographs of composites with varied reinforcement content. Higher percentage reinforcement, on the other hand, resulted in particle aggregation. In a few places, the microstructure of ASC and ASSC composites included clusters of SiC, and this agglomeration happened due to SiC has a higher density (3.20 g/cm^3^) than aluminum (2.67 g/cm^3^). This is owing to the fact that the high-density SiC particles have a tendency to sink to the bottom of the matrix as a result of gravitational forces, which might result in areas of higher concentration and agglomeration^38,39^. Optical micrograph (Fig. 6) clearly indicates about agglomeration and it is shown with dotted area.

The lower density of B_4_C particles with compared to SiC assists in a greater scattering of particles with the matrix, resulting in less agglomeration of B_4_C particles in the ABC and ABBC composite. Both composites (B_4_C and SiC reinforced) have a microstructure of extremely partial voids, indicating strong interfacial bonding between matrix (aluminum) and reinforced materials (boron carbide, Silicon carbide, and CNT). The continual stirring and squeeze action used to achieve a homogenous mixture, and also helped to prevent oxide formation and intermetallic (Al_3_BC and Al_4_C_3_) phase formation. During the solidification of the composites, the particles settle uniformly inside the molten matrix. The visual assessment of the microstructure indicates the presence of blowholes and shrinkage. In the microstructure, shrinkage shows as pores or voids, whereas blowholes appear as gas pockets or cavities. In addition to visual inspection, metallography can make it easier to estimate the size and distribution of the voids, which can assist, how they can affect the casting's mechanical properties. The microstructural analysis revealed no such major undesirable phase or casting defects formed such as shrinkage, blowholes, or pits.

### TEM analysis of the composite

**Figure 8:**
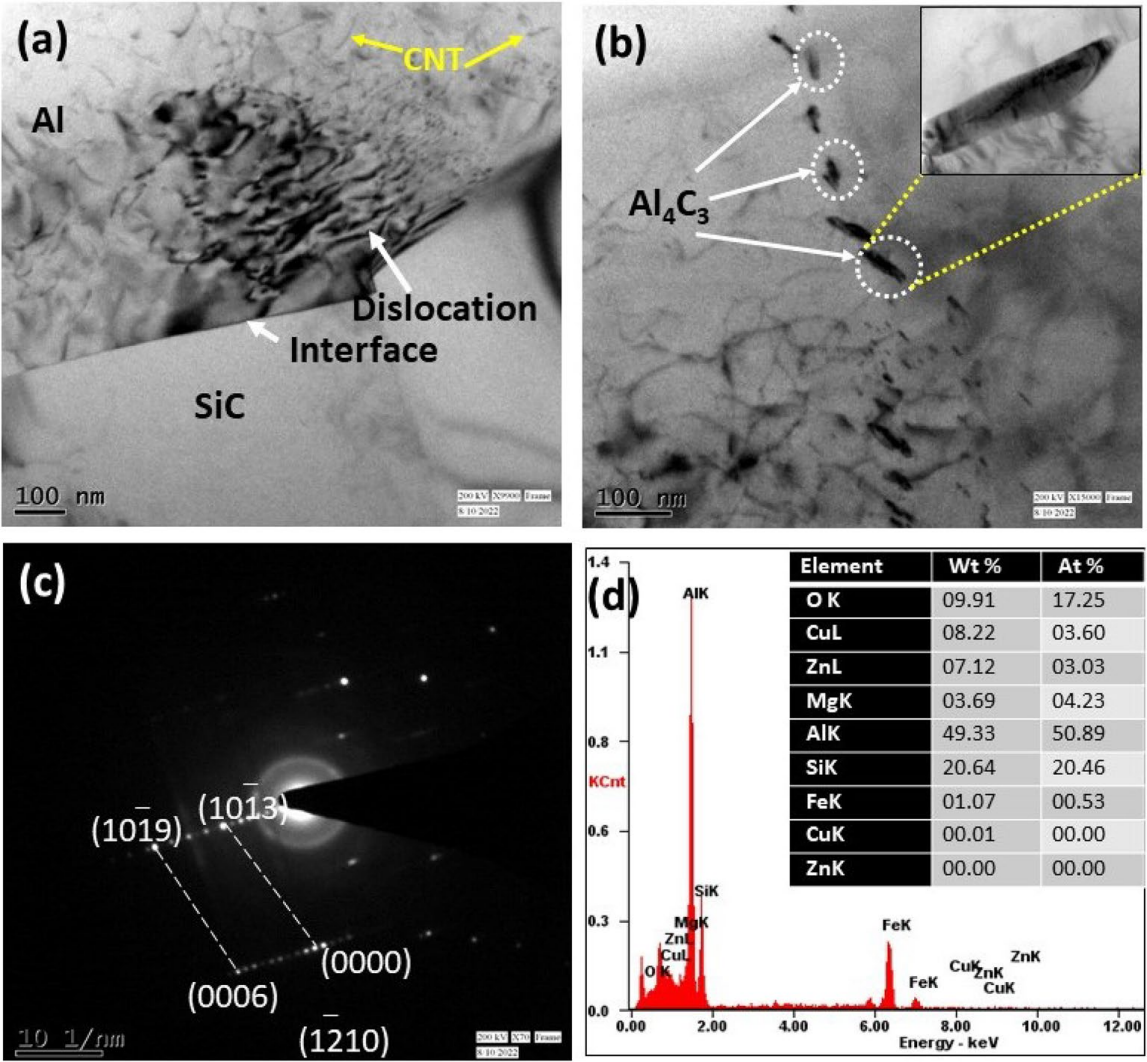
TEM morphological analysis for ASSC in the view of (**a**) matrix and CNT, SiC presence, (**b**) precipitation formed as Al_4_C_3_, (**c**) selected area electron diffraction EDS pattern, and (**d**) TEM EDS analysis.

**Figure 9:**
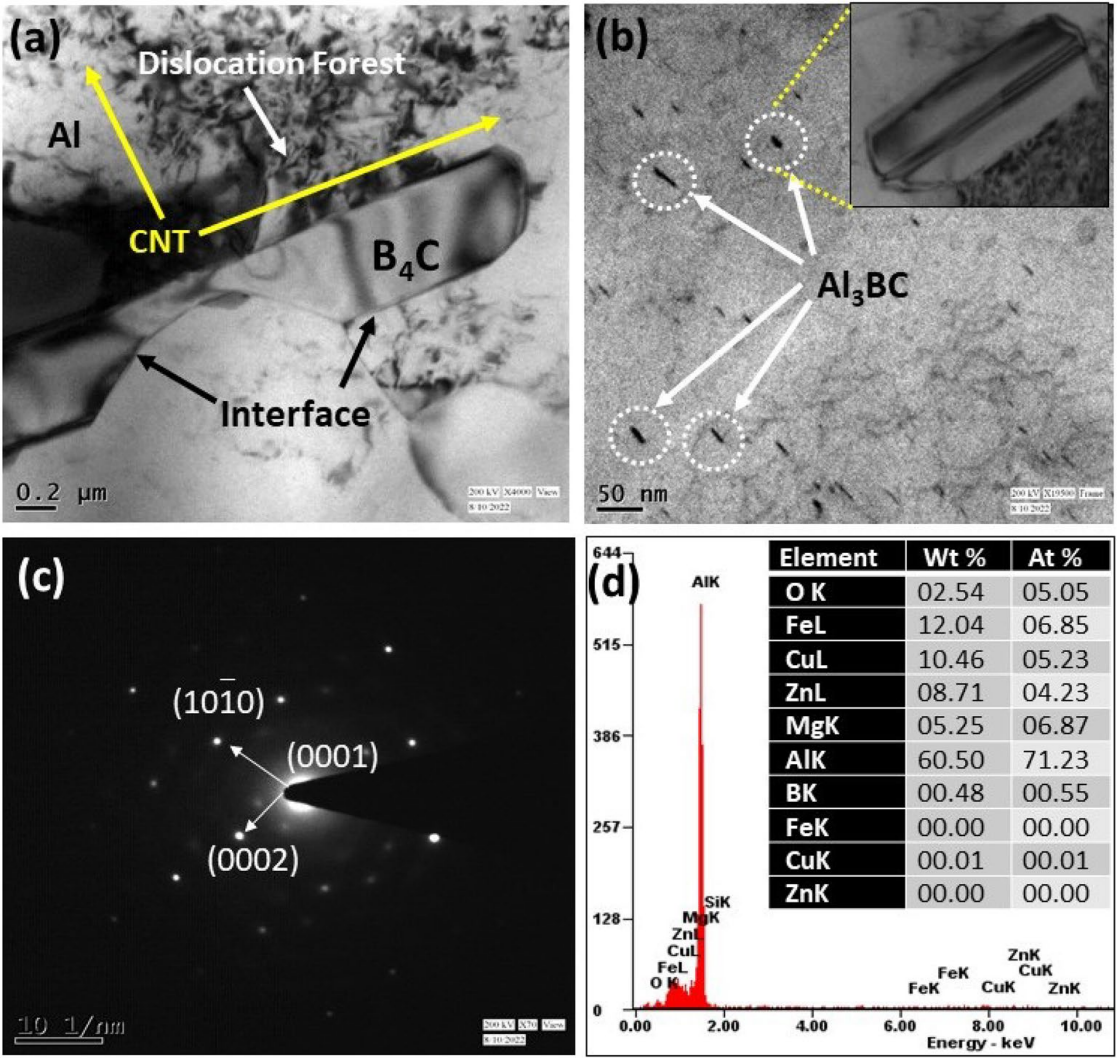
TEM morphological analysis for ABBC in the view of (**a**) matrix and CNT, B_4_C presence, (**b**) precipitation formed as Al_3_BC, (**c**) Selected area electron diffraction EDS pattern, and (**d**) EDS analysis.

The ability of a transmission electron microscope to provide high-resolution micrograph up to the level of atomic structure of materials makes it a useful tool for material characterization. To visualize the material's microstructure and get insight into its characteristics, high resolution pictures taken using transmission electron microscopy are necessary for aluminum metal matrix composites. Observations were done using a TEM to more thoroughly confirm the microstructure of the tested composites. Selected findings are shown in Figs. 8 and 9. The presence of sub-grains near the matrix material is a defining property of the substructure of the materials under study (Figs. 8a, 9a). The sub-grains are smaller than 1 μm in size. Locally, Al_4_C_3_ clusters and dislocations forming low-energy systems are seen Fig. 8b. Al_4_C_3_ particles are no larger than 50 nm in size. The carried out TEM microstructure analyses identified an undesirable carbide, and it is shown in Figs. 8b and 9b. The research on casting composites made of Al-SiC at 850 °C^40^, contains attempts to explain how this combination formed. The Al_4_C_3_ brittle compound and free silicon particles develop at the boundary between these two phases as a result of the interaction between liquid Al and SiC particles, according to the authors of^2,41–43^, which negatively affects the mechanical characteristics. The molten Al reacted with SiC, and forms Al_4_C_3_ Eq. (3)^40,44^:

3 $${\text{Al }} + {\text{ SiC }} \to {\text{ Al}}_{{4}} {\text{C}}_{{3}} + {\text{ Si}}$$

The B_4_C reinforcements and the aluminum matrix underwent interfacial reactions as the mixing temperature rose to 850 °C. A B_4_C particle is shown in Fig. 9a embedded in the composite. The B_4_C/matrix contact shows a different shape from that in Fig. 7a. At the contact, a layer of reaction byproducts made up of many nanoparticles emerged continuously. The TEM image depicted in Fig. 9b is a single nanoparticle. The particles are Al_3_BC crystals formed, according to the SAED (selected area electron diffraction) pattern inset in the picture. The B_4_C/Al reactions also led to the development of a brittle phase or high density of nanoscale precipitates in the composite, which were accompanied by Al_3_BC particles and it formed from 578 to 645 °C^45^. The analysis of the composite that was created above 645 °C shows that the precipitates underwent a significant alteration as a result of the interfacial processes. Specifically, Al_3_BC and other boron-containing reaction products. It is appropriate to attribute the process to the chemical processes that were made possible by the advent of molten aluminum. The responses are as follows with chemical reaction Eq. (4):

4 $${\text{Al }} + {\text{ B}}_{{4}} {\text{C }} \to {\text{Al}}_{{3}} {\text{BC}} + {\text{B}}$$

Specifically, reaction (2) took place when the B_4_C and Al melt reacted into one another at the interface boundaries. Figures 8c and 9c illustrate the composite's SADP (selected area diffraction patterns) and it is obtained from TEM analysis and the findings revealed just one set of diffraction patterns in both SADP pictures. Figure 8c shows the zone axis for Al_4_C_3_, which is $[1\overline{2 }10]$, while Fig. 9c shows the same zone axis for Al_3_BC, which is $[10\overline{1 }0]$. Figures 8d and 9d show the TEM EDS, which confirms the existence of intermetallic formation in boron carbide and silicon carbide reinforced composites.

The majority of studies have seen Al_3_BC as a reaction product in B_4_C reinforced AMCs^46–48^. This reaction also produced free boron in addition to Al_3_BC, which later diffused into the aluminum matrix. There has been an increase in interest in the solute atom interfacial segregation that has been seen in numerous aluminum alloys recently. A recent study by Fiawoo et al.^49^ demonstrated, for instance, Cu segregation at Q/Al contacts in an Al–Mg–Si–Cu alloy.

Other findings include the segregation of Mg at Al_3_Sc/Al surfaces in Al–Mg–Sc alloys^50^, Si at Q/Al interfaces in Al–Si–Cu alloys^51^, and Mg–Ag–co at U/Al interfaces in Al–Cu–Mg–Ag alloys^52^, among others. Magnesium alloys have also shown similar solute segregation at hetrophase interfaces^53,54^. Although several theories have been put out to explain interface segregation, it is generally agreed upon that solute agglomeration can lower interfacial energy^51,55^. The precipitation's nucleation rate and coarsening resistance would both increase when the interfacial energy was reduced. The species, shapes, and sizes of the interfacial reaction products are thought to have a substantial impact on how loads are transferred through the aluminum matrix and reinforcements, which surely affects the mechanical behavior of the metal matrix composite^56^. Therefore, it is key investigation to continue, how micro-alloying affects the mechanical characteristics of AMCs.

### Elemental mapping of the composite

**Figure 10:**
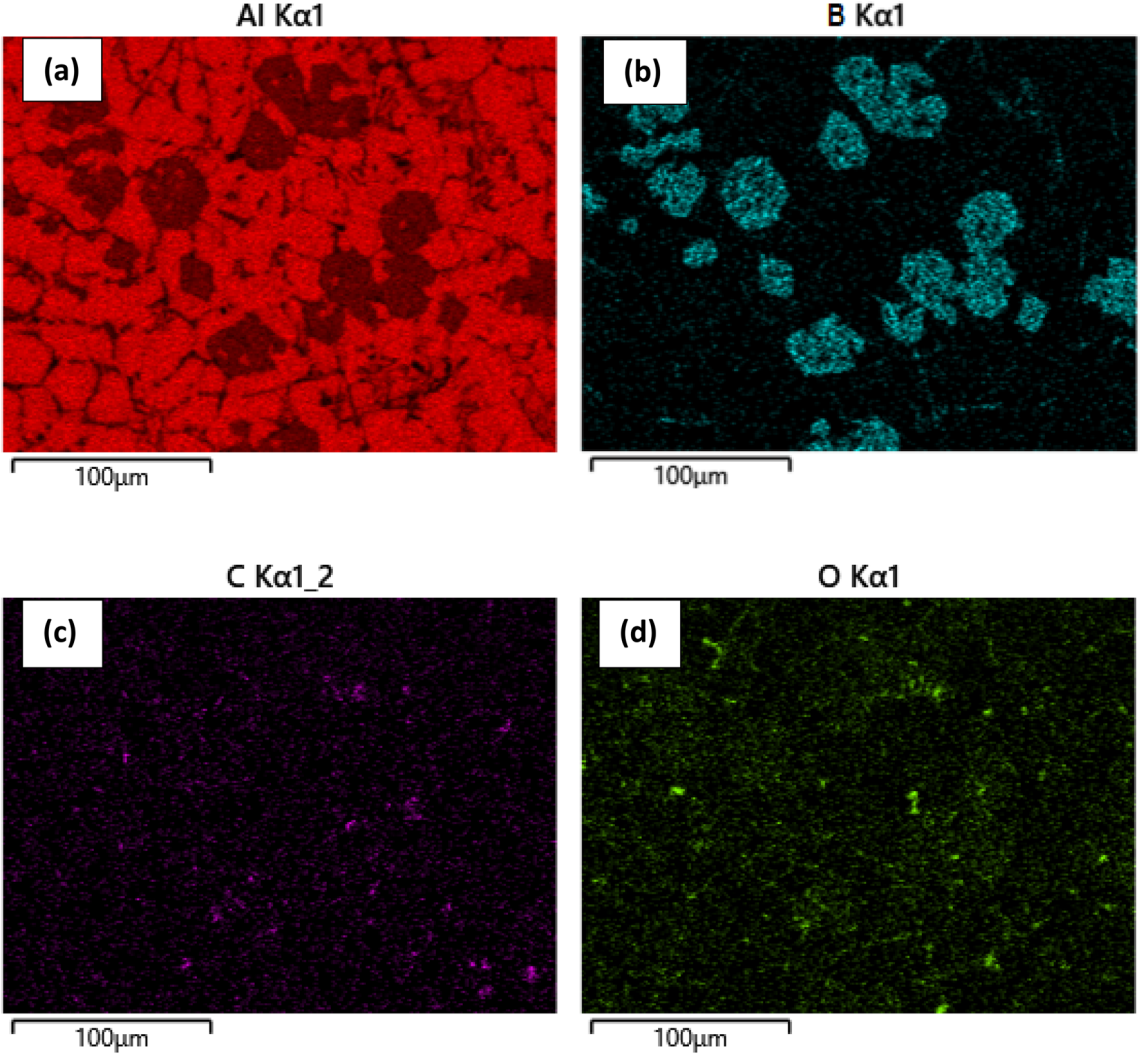
Elemental mapping indicates (**a**) aluminum (Al), (**b**) boron (B), (**c**) carbon (C) and (**d**) oxygen (O) in B_4_C reinforced composite.

**Figure 11:**
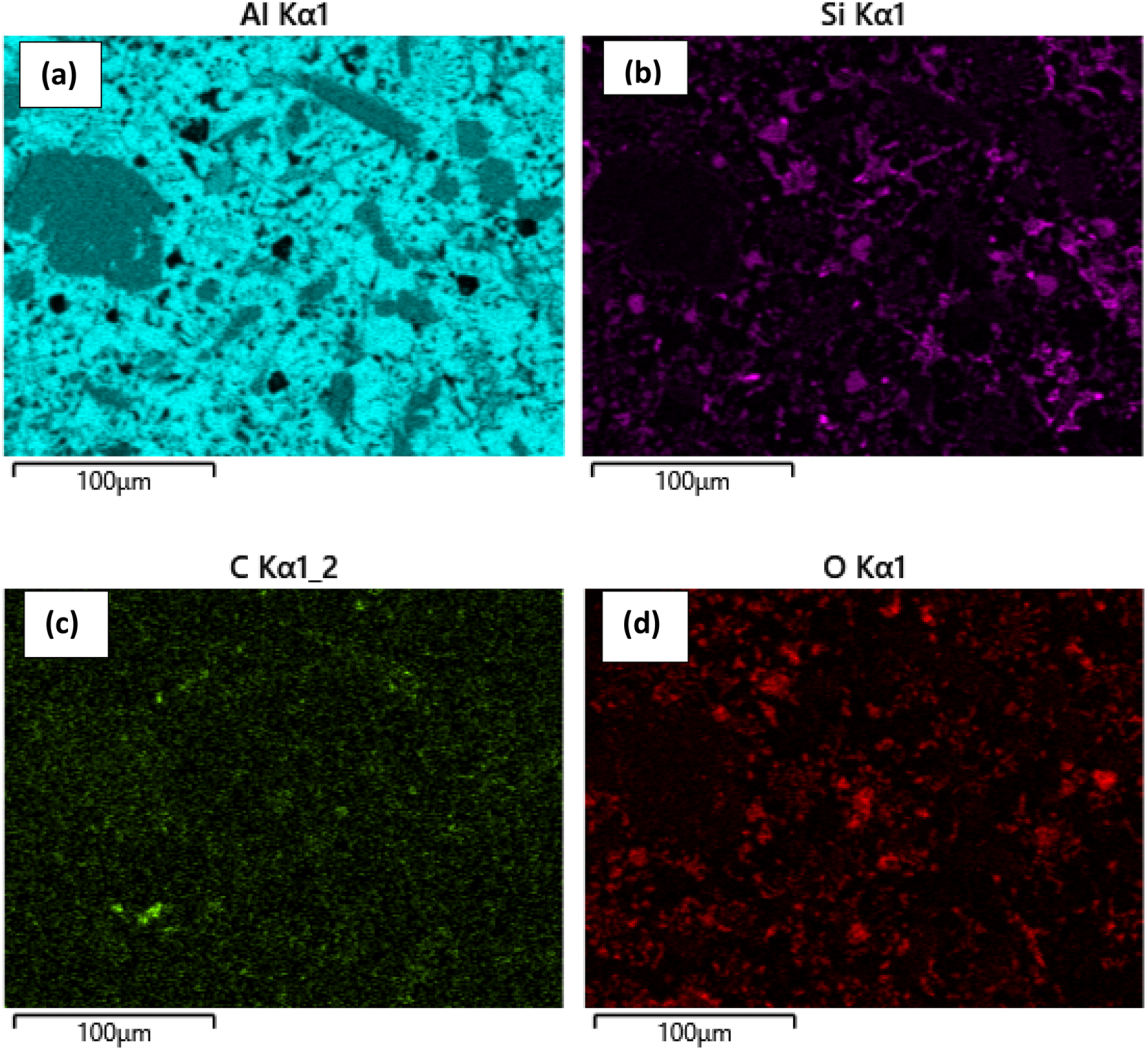
Elemental mapping indicates (**a**) aluminum (Al), (**b**) silicon (B), (**c**) carbon (C) and (**d**) oxygen (O) in SiC reinforced composite.

The composite is made with an aluminum matrix and two types of reinforcements: (B_4_C + CNT) and (SiC + CNT). Since, CNT has a large surface area, agglomeration is more likely than a uniform distribution throughout the volume. The EDX mapping was used to confirm that the reinforcement was distributed evenly. The EDX mapping of the Al hybrid composites is shown in Figs. 10 and 11, and it is obvious that MWCNTs, B_4_C, and SiC are widely disseminated inside the composites.

This demonstrates that reinforcements are dispersed more successfully in the Al matrix, despite the hybrid system's inclination to cluster. It is well understood that effective MWCNT cluster dispersion within the Al matrix likely to much difficult, preventing the creation of pores and the development of micro-cracks in composites to some extent. Figure 10a–d depict the presence of aluminum, boron, carbon, and oxygen, respectively. Similarly, Fig. 11a–d depict aluminum, silicon, carbon, and oxygen, respectively. The presence of spots in Figs. 10c and 11c are carbon throughout the volume, indicating that MWCNTs are distributed uniformly. Some oxide is formed during casting and is visible in both composites, but it has little impact on the properties. Similarly, boron for B_4_C and Si for SiC have been seen in Figs. 10b and 11b, respectively, and are uniformly distributed.

### Micro-mechanical analysis

The investigation of properties at the nanoscale is a little difficult, but it saves materials for specimen preparation. Lower volume of materials required is strong evidence for lower cost experimentation, which is a much more economical testing methodology than the traditional mechanical testing method. For composite materials, it is necessary to investigate micro-mechanical characteristics in order to comprehend the performance of a composite material at the nanoscale level, which can shed light on the material's overall performance. The nano indentation test method of the composite results in the conclusion of several desired properties at the nano scale. Mechanical characteristics including hardness, elastic modulus, and fracture toughness are measured by nano indentation at the nanoscale in composite materials.

#### Young’s modulus

**Figure 12:**
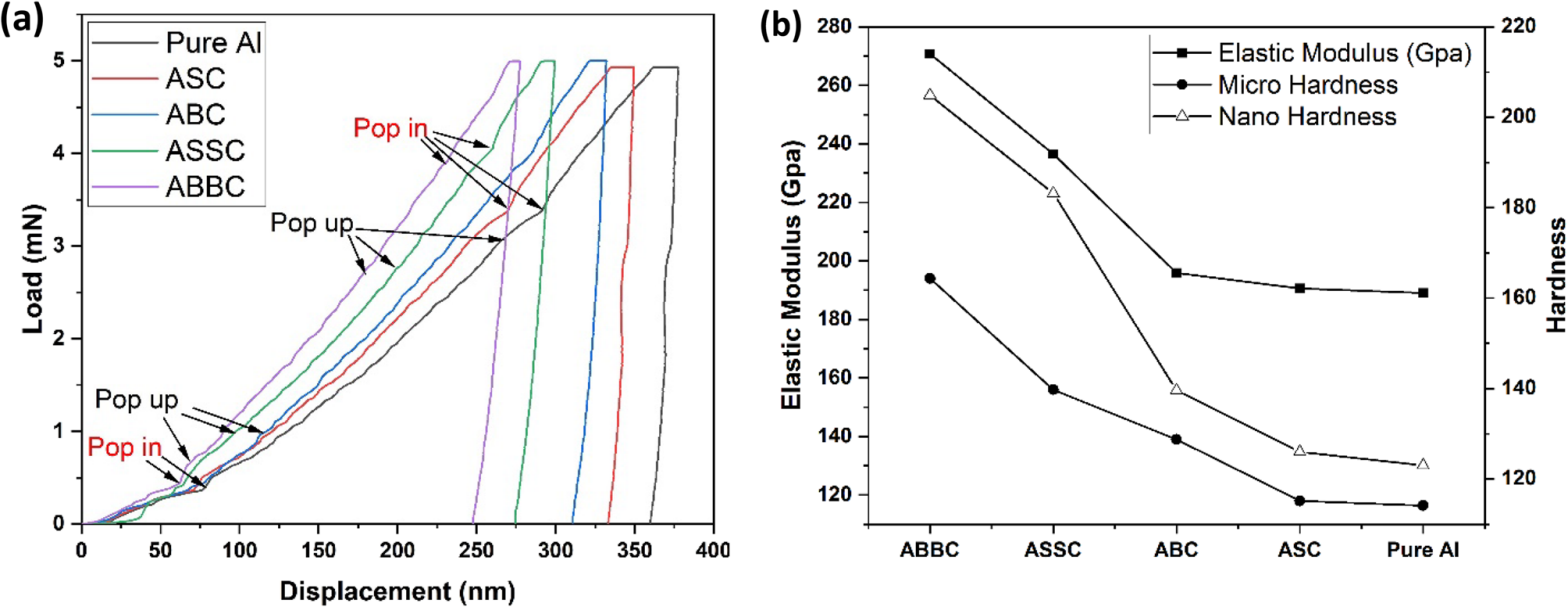
(**a**) Load–displacement curve for composite (**b**) hardness (micro and nano) and modulus of elasticity.

The composite's (ABBC, ABC, ASSC, and ASC) load–displacement curve has been exhibited and it is shown in Fig. 12a. The reduced Young's modulus (${E}_{r}$) is used to calculate Young's modulus (E) of the composite, and it is obtained from Nano indentation test of the composite by using Eq. (5)^57^:

5 $$\frac{1}{{E}_{r}} =\left(1-\frac{{{V}_{s}}^{2}}{E}\right)+\left(1-\frac{{{V}_{i}}^{2}}{{E}_{i}}\right)$$

where ${V}_{s}$ (0.3) and ${V}_{i}$ (0.07) is the Poisson ratio of specimen and indentation, respectively. ${E}_{i}$ (1140 Gpa) is the indentation of Young’s modulus.

The load–displacement curves of both boron carbide and silicon carbide are shifted to the left, indicating that reinforcement has strengthened the matrix. When compared to ABC, ASSC, and ASC composites, ABBC shows the least displacement, indicating that ABBC has the highest modulus of elasticity. According to the load–displacement (nano depth) curves, ASC has the greatest displacement and thus the lowest Young's modulus. The same evidence has been seen in Fig. 12b for the composite’s modulus of elasticity. The composite with silicon carbide or boron carbide reinforcement displays relatively reduced penetration depth, and more reinforcement leads to less penetrating displacement in the composite. However, the boron carbide reinforced composite exhibits the least displacement. The composite ABBC, ASSC, ABC, and ASC have Young's moduli that are 41%, 20% and 5% and 2% higher than pure Al, respectively.

Young's modulus of ABBC is higher than ASSC due to less agglomeration, and the boron carbide and CNT show good agreement with the matrix, as seen in SEM images also. Furthermore, the greater Young's modulus indicates effective load transfer between reinforced particulates and the aluminum matrix. Among the ABBC, ASSC, ABC, and ASC composites, the ABBC composite has the highest modulus. The load displacement curve also includes pop-in and pop-up events, which help to understand the influence of the pop-in and pop-up events on different compositions. The commencement of plastic deformation is linked to the pop-in and pop-up phenomena on the materials, and this combination has been discovered more frequently in the case of ABBC and ASSC composites, and extremely seldom in ABC, ASC and pure Al.

#### Shear lag model

The shear lag theory was also used to estimate Young's modulus, which was then compared to experimental data (nano-indentation). In this concept, load is transmitted from the matrix to the reinforcement via interfacial shear stress. According to this model, the composite's young's modulus (${E}_{c}$) is calculated using Eq. (6)^58^:

6 $${E}_{c}=f{E}_{f}\left[1-\frac{\mathrm{tanh}\left(ns\right)}{ns}\right]+\left(1-f\right){E}_{m}$$

7 $$n=\sqrt{\frac{2{E}_{m}}{\left[{E}_{f}\left(1+{V}_{m}\right)\mathrm{ln}\left(\frac{1}{\mathrm{f}}\right)\right]}}$$

where reinforcement fraction ($f$), Young’s modulus of matrix (${E}_{m}=68\times {10}^{9}\frac{\mathrm{N}}{{\mathrm{m}}^{2}}$) and aspect ratio ($s=100$), matrix Poisson ratio $( {V}_{m}=0.32)$ and ${E}_{f}$ is the complex Young’s modulus of the particulate and it can be calculated for two-component composite by using Eq. (8)^59^:

8 $${E}_{f}={v}_{1}{E}_{1}+{v}_{2}{E}_{2}$$

${v}_{1}$ and ${v}_{2}$ is the volume fraction of the particulate used in the composite and ${E}_{1}$ and ${E}_{2}$ is Young’s modulus of the particulate (there were two sets of reinforcement (B_4_C + CNT) and (SiC + CNT)) in Eq. (4). $n$ have been calculated by using Eq. (7) for Young’s modulus of the aluminum metal matrix composite.

#### Isostrain model

The composite's Young's modulus is also predicted using the isotrain model by using Eq. (9)^60^. The isostrain model takes into account uniaxial loading over the fiber length, with equal strain in the fiber, matrix, and composite.

9 $${E}_{c}={E}_{m}{v}_{m}+{E}_{f}{v}_{f}$$

Here ${E}_{c}$, ${E}_{m}$ and ${E}_{f}$ is the composite, matrix, and fiber/particulate’s Young’s modulus and ${v}_{m}$ and ${v}_{f}$ are the volume fraction of matrix and particulate in the aluminum matrix composite.

For the nanocomposite ABC, ABBC, ASSC, and ASC, respectively, a rise in Young's modulus of 19% and 39% was discovered, and it is very closely resembling the results from the shear lag model.

#### Isostress model

In this model, equal stresses in the composite, matrix, and fibers are examined under uniaxial loading along the fiber's length in the transverse direction. According to this model, adding MWCNTs has no discernible effect on the modulus.

10 $$\frac{1}{{E}_{c}}= \frac{{v}_{f}}{{E}_{f}}+\frac{{v}_{m}}{{E}_{m}}$$

Equation (10)^60^ is used to calculate the composite's Young's modulus where, ${E}_{c}$, ${E}_{m}$ and ${E}_{f}$ is the composite, matrix, and fiber/particulate’s Young’s modulus and ${v}_{m}$ and ${v}_{f}$ are the volume fraction of matrix and particulate in the aluminum matrix composite.

**Figure 13:**
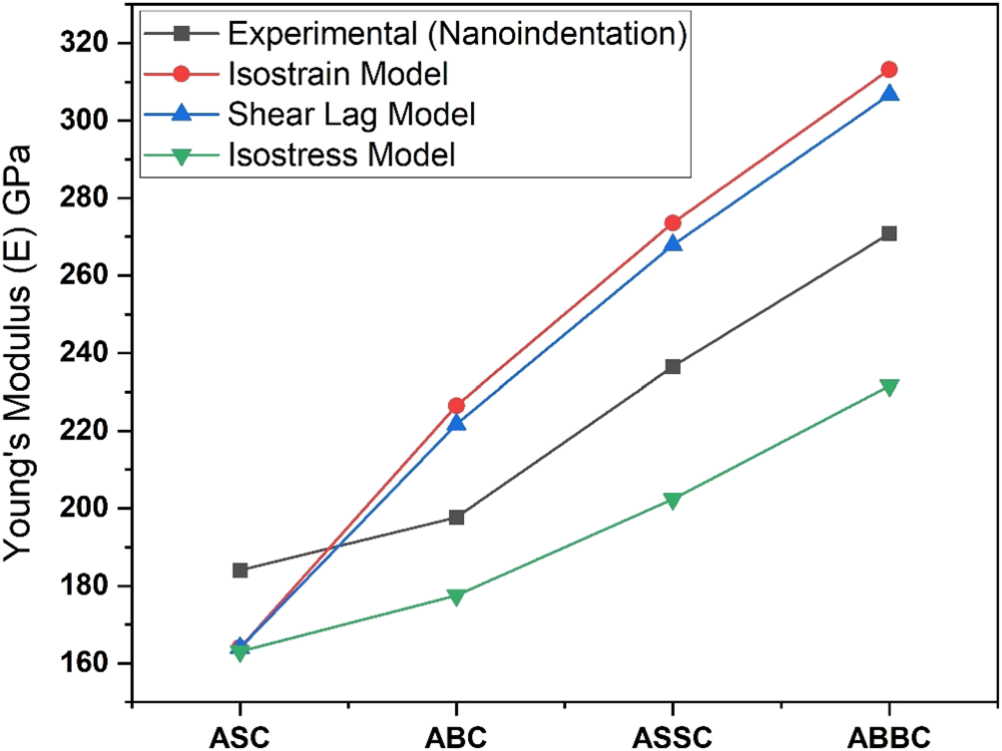
Comparative young’s modulus of experimental and different model.

Figure 13 shows a comparison of Young's modulus, and it is driven by the shear lag, isostrain, and isostress models of the composite. The isostrain and shear lag models should be noted and it is overestimate Young's modulus values, whereas the isostress model underestimates them. The nanocomposite deviates from the isostrain model's assumption that stresses in the fiber, matrix, and composite are equal. As a result, the isostrain model overestimates rather than predicts real values. The isostress model also makes the false assumption that the stresses in the fiber, matrix, and composite are all the same. As a result, this model underestimates rather than predicts real values. Interfacial shear stress is used in the shear lag model to transmit stress from the matrix to the fiber. It also presupposes that the matrix and fiber are perfectly bonded, which is not the case. As a result, this model underestimates rather than predicts real values.

#### Micro and nano hardness

Hardness has a significant role in deciding how a composite material will behave mechanically. Harder materials tend to be more resistant to wear and abrasion, while softer materials tend to be more ductile and able to absorb more energy. Hardness can also affect the fatigue life of a material; as harder materials tend to have longer fatigue lives. In terms of hardness, two forms of hardness have been studied: micro hardness (Vicker's hardness) and nano hardness (nano indentation). Equations (1) and (11) are used to calculate micro and nano hardness, respectively. Using nano indentation to make small indentations on the specimen's surface, the composite's nano hardness was measured, and the load was calculated using Eq. (11)^61^.

11 $$H=\mathrm{P}({h}_{max})/{A}_{c}({h}_{max})$$

where P is the applied load and ${A}_{c}({h}_{max})$ is the instantaneous contact area between the material and the indenter. The volume fraction of B_4_C/SiC and MWCNTs was shown to improve the nano hardness. Hardness was calculated using the maximum load in the load–displacement curve.

Table 4 shows the comparative nano and micro-hardness of the aluminum nanocomposite. In comparison to micro-hardness, nano hardness results revealed an increase in hardness. When doing a nano indentation test, the hardness was evaluated at the lowest load and smallest indentation area. Micro-hardness takes into account the indented area following indentation, which may or may not be correct due to elastic recovery after indentation. Furthermore, the high-reinforced samples had a higher hardness than the lower-reinforced samples, showing that effective load transfer occurs between the matrix and the particulate, assuring effective reinforcement.

**Table 4:** The NANO hardness and Vickers hardness of the specimen.

S.no	Fabricated specimen	Nano hardness (GPa)	Vickers hardness (HV_0.1_)
1	ABBC	204.9 ± 5	194 ± 2
2	ASSC	183.14 ± 3	156 ± 3
3	ABC	139.64 ± 6	139 ± 1
4	ASC	126.01 ± 4	118 ± 1
5	Pure Al	104.09 ± 5	91 ± 3

**Figure 14:**
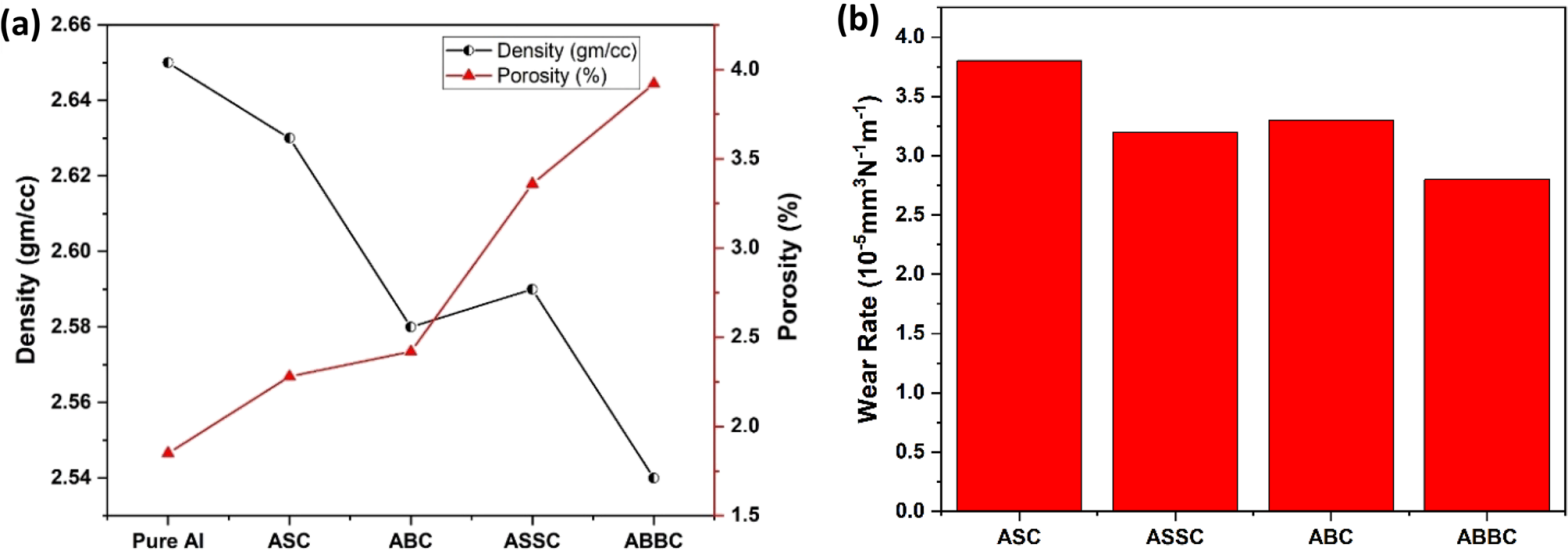
(**a**) Variation of density and porosity (**b**) comparative wear rates of the composite.

Figure 14a shows the density and porosity of the composite. Porosity increases inevitably cause a reduction in density. For ABBC composite, the porosity rises from 1.8 to 3.9% when compared to pure aluminum. ABBC has a 1% greater porosity than ASSC. A composite material's density can have a significant impact on its mechanical characteristics, including its toughness, stiffness, and strength. Higher density materials have greater strength and stiffness, while lower density materials have greater toughness. When the reinforcement of micro and nano particles in the matrix grows, the porosity of the matrix increases, resulting in a decreased density of the composite. This happened as a result of the fine particles' high surface area. The higher reinforcement, greater porosity and lower density of the composite. In such dependent properties, the mechanical properties of ABBC should be the lowest, however the outcome is entirely different. And this occurred because the reinforcement stopped the grain boundary from slipping as the crack was propagating. If the crack's velocity of spread slows down, the fracture may take longer to occur and result in a greater modulus of elasticity. Figure 12b illustrates this as well.

### Tribological behavior of the composite

The wear rates of the composite with varied B_4_C and SiC concentrations are shown in Fig. 14b. As can be observed, changing the reinforcing content significantly affects the composite's wear rate. The wear rate of the composite is obtained lower comparatively, when the SiC and B_4_C content is high, which is likely due to the fact that the SiC and B_4_C particles are employed to hinder the plastic deformation throughout the composite and higher amount more likely to resist. The nano reinforced effect is also apparent, which also contributes to the reduced wear rate. The wear resistance of the composite is, nevertheless, comparatively low when the concentration of B_4_C and SiC is low, and it is high when reinforcement particulates is higher in the composite. However, when the B4C concentration reaches 12 vol%, the composite's wear rate and friction coefficient concurrently achieve their lowest and maximum values. This shows that the composite's performance at this component ratio is optimal, which may be because B_4_C and CNT are evenly distributed throughout the composite. It is interesting to note that as the friction coefficient rises, anti-wear properties are concurrently increases from 2.7 × 10^–5^ mm^3^ N^−1^ m^−1^ to 3.8 × 10^–5^ mm^3 ^N^−1^ mm^−1^ (raised by 40%) of high reinforced composite. ASC has the highest wear rate recorded, whereas ABBC had the lowest wear, with a difference of nearly 40%. The wear rates for ASC and ASSC are 3.8 × 10^–5^ mm^3 ^N^−1^ mm^−1^ and 3.3 × 10^–5^ mm^3 ^N^−1^ mm^−1^, respectively. The comparable tendency has been seen for ABC and ABBC as 3.4 × 10^–5^ mm^3^ N^−1^ mm^−1^ and 2.7 × 10^–5^ mm^3 ^N^−1^ mm^−1^, respectively.

**Figure 15:**
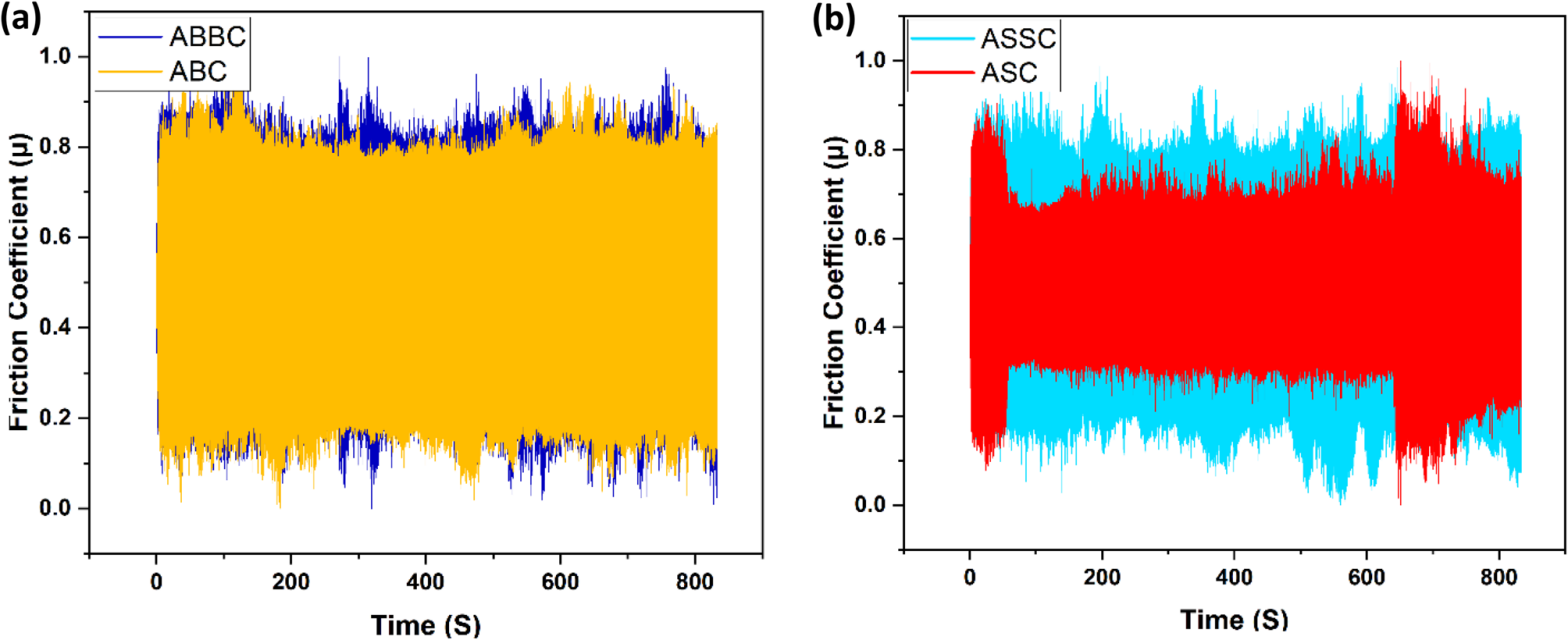
Variation of friction coefficient vs time of (**a**) B_4_C reinforced (**b**) SiC reinforced nanocomposite.

The friction coefficient curves of the composite with different reinforcing amounts are shown in Fig. 15a,b. Due to the fact that all friction coefficients exhibit a constant and decreasing level over the course of the measured time period, it is obvious that the composite has a lower friction coefficient and that the changing trend during the rubbing procedure is more consistent, when the B_4_C amount is 12 vol%. In order to achieve desired wear behavior and increase the durability of composite materials, it is crucial to understand the impact of friction coefficient on wear behavior of a composite material. Because boron carbide reinforcement has a more consistent friction coefficient, a better level of wear resistance is attained.

**Figure 16:**
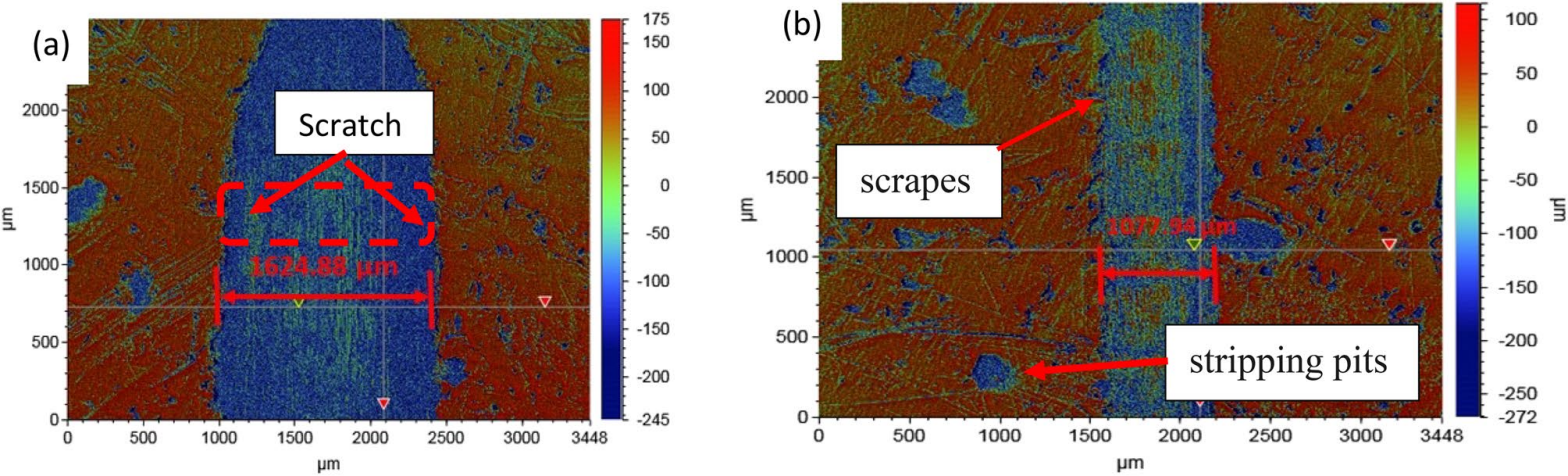
2D scratch view of the surface of (**a**) ASSC (**b**) ABBC composite.

**Figure 17:**
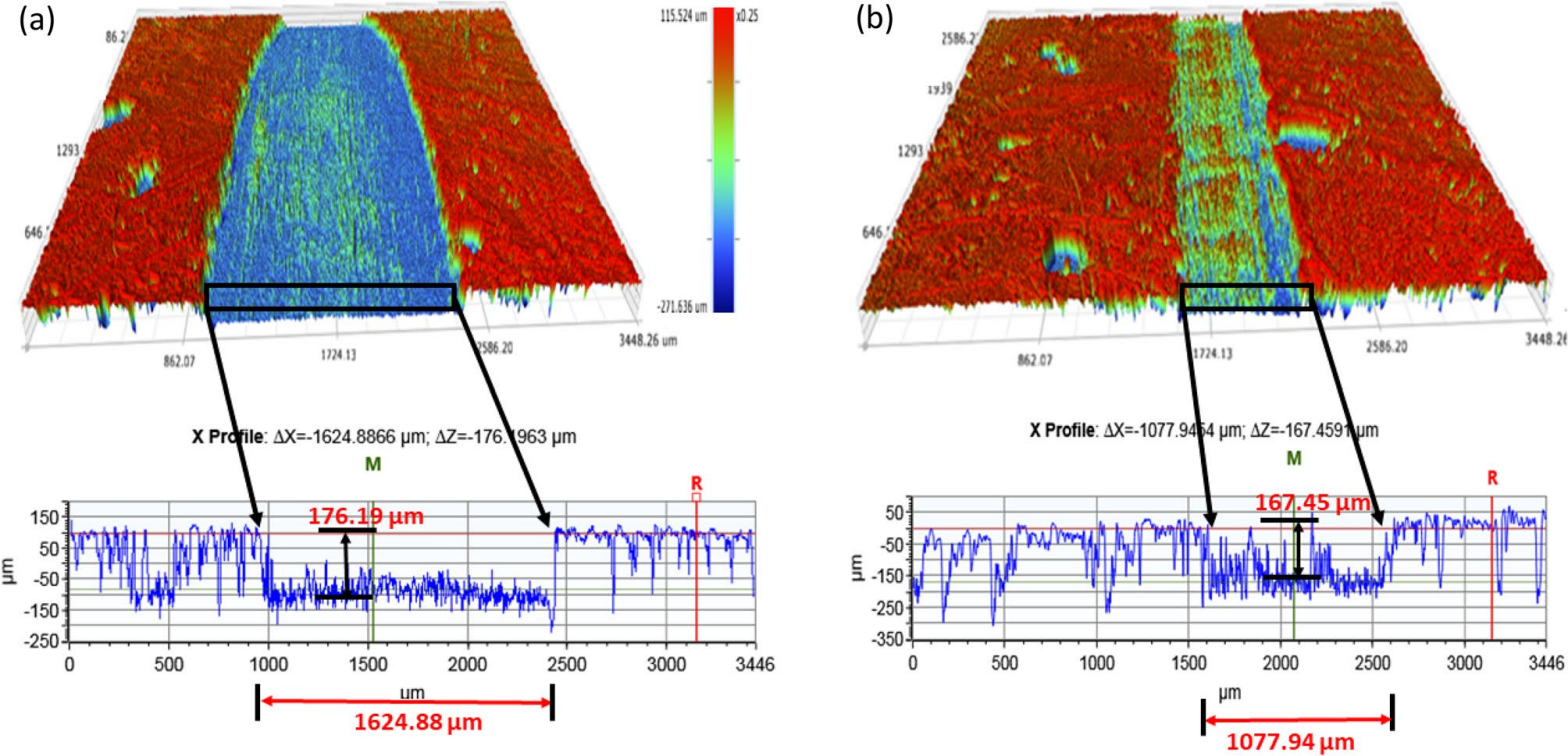
(**a**) Topography of the scratched surface for ASSC with X profile (ΔX = 1624.8866 µm; ΔZ = − 176.1963 µm) and (**b**) scratched surface for ABBC with X profile (ΔX = 1077.9454 µm; ΔZ = − 167.1963 µm).

Studies of topography entail analyzing a surface to ascertain its characteristics and texture. This kind of research may be utilized to distinguish between a composite material's abrasion behavior since varied textures and characteristics can have an impact on how the material reacts to wear and tear. Optimizing the design and functionality of the materials used in applications with high wear and abrasion activity, topographical studies are needed for aluminum metal matrix composites. These studies help to comprehend the surface features of the composite material, which may have applied for automotive application. The characterization features serve as a better decision-making tool for the development of materials for the automotive or aerospace industries. The wear rate of the contacting dual can further support the composite's tribological characteristics^62,63^. Figures 16 and 17 demonstrate, respectively, the wear scars and 2D and 3D surface topography of composite specimen surface with varying volume percentages of B_4_C and SiC. The smaller diameter for wear scar has observed, when the mass fraction of B_4_C is 12 vol%, as can be perceived from the surface topography. When the SiC concentration is high, there are more wear scars and furrow scratches on the surface of the composite specimen. The degree of wear of the composites by virtue of wear scar and furrow scratches differs significantly, as seen in Fig. 16b.

When SiC reinforcement is 12 vol%, the wear width and depth are significant and Within the worn markings are a number of scrapes and stripping pits. The wear width and depth along with less scratch and stripping pits observed, when it is reinforced with B_4_C of 12 vol% in the matrix. This is when, it is compared to other reinforcement levels, and it indicates the composite is safe from severely damaged because high pits and broader scratches is indication for severe damage.

Because of the agglomeration effect in composites, the amount of reinforcement is limited. The agglomeration effect is reduced by modifying the manufacturing procedure, which is done in the current study effort. When compared to SiC reinforcement, B4C reinforced composite has improved mechanical behaviour. However, increased reinforcing in both types of composites has resulted in improved properties. Mechanical characteristics have a direct influence on the surface behaviour of the composite.

The study mentioned above leads to the conclusion that the wear scar has an even distribution of all the composite's components. Therefore, significantly increasing the composites' ability to reduce friction and withstand wear. The composite hence has outstanding tribological characteristics.

During various sliding cycles, some published study examined changes in the related coefficients of friction, the wear marking’s track and wear volume to analyze the variation laws of the composite's tribological features^64–66^. To comprehend the composite's failure process better, It is crucial to thoroughly examine how the friction coefficient, wear volume, and wear mark morphology are related. Reinforcement materials in the matrix may have contributed to this outcome. It will be simpler for SiC and B_4_C to create a continuous lock of the rubbing face and penetrate into the matrix interface, which can reduce shearing stress and result in minimal wear^67^.

The experiment findings indicate that the reinforcement has a momentous impact on the manufacturing of aluminum hybrid nano composites; nevertheless, the fundamental physical, chemical, and tribological characteristics of the 12 vol% B_4_C reinforced composite exhibit inflective with SiC reinforcement. Among the many causes, the noticeable improvement in tribological characteristics of the B_4_C-CNT based composite is attributed to the suitable incorporation of reinforcement, which increases strength. Otherwise, the variation laws underlying the tribological phenomena were investigated by the sliding cycle is being increased and altering the investigational parameters (the load being exerted while sliding) to study the variation in wear rates and friction coefficients, with the following results: the wear of the composite occurs mostly during the early phase of abrasion, and once the sliding cycle has passed the run-in stage, the friction coefficient and wear volume are not affected by changes in the sliding cycle. Moreover, the wear rates show a strong positive association with the applied load, although the average friction coefficients drop with increasing applied load. The pace at which the composite wears out increases with increasing load. The wear behavior of aluminum composites may also be learned via scratch testing. A scratch causes surface degradation by introducing localized deformation and forces that cause wear debris. By changing the contact area and stress concentration, the depth and width of the scratch can change the wear behavior. In general, the severity and distribution of mechanical loads as well as the building of temperature are influenced by the friction coefficient and scratch, which in turn has an impact on how aluminum composites wear. When analyzing wear behavior, it is important to take into account the composite material's mechanical and microstructural characteristics as well.

The results of the studies showed how important it is to consider other surface characteristics, in addition to the 2D parameter, which is frequently provided in standards, such as surface roughness parameters (3D parameters), the Abbott-Firestone curve, and its parameters, which could all be important when interpreting the findings of tribological studies. The exhibiting surface morphology are the only things that researchers are primarily concerned with, therefore studies on manufactured surface topographies are typically overlooked or merely skimmed through (pictures generated using SEM or OM). It is essential to identify any surface modification treatment of the materials in order to obtain the better working life of the materials.

**Figure 18:**
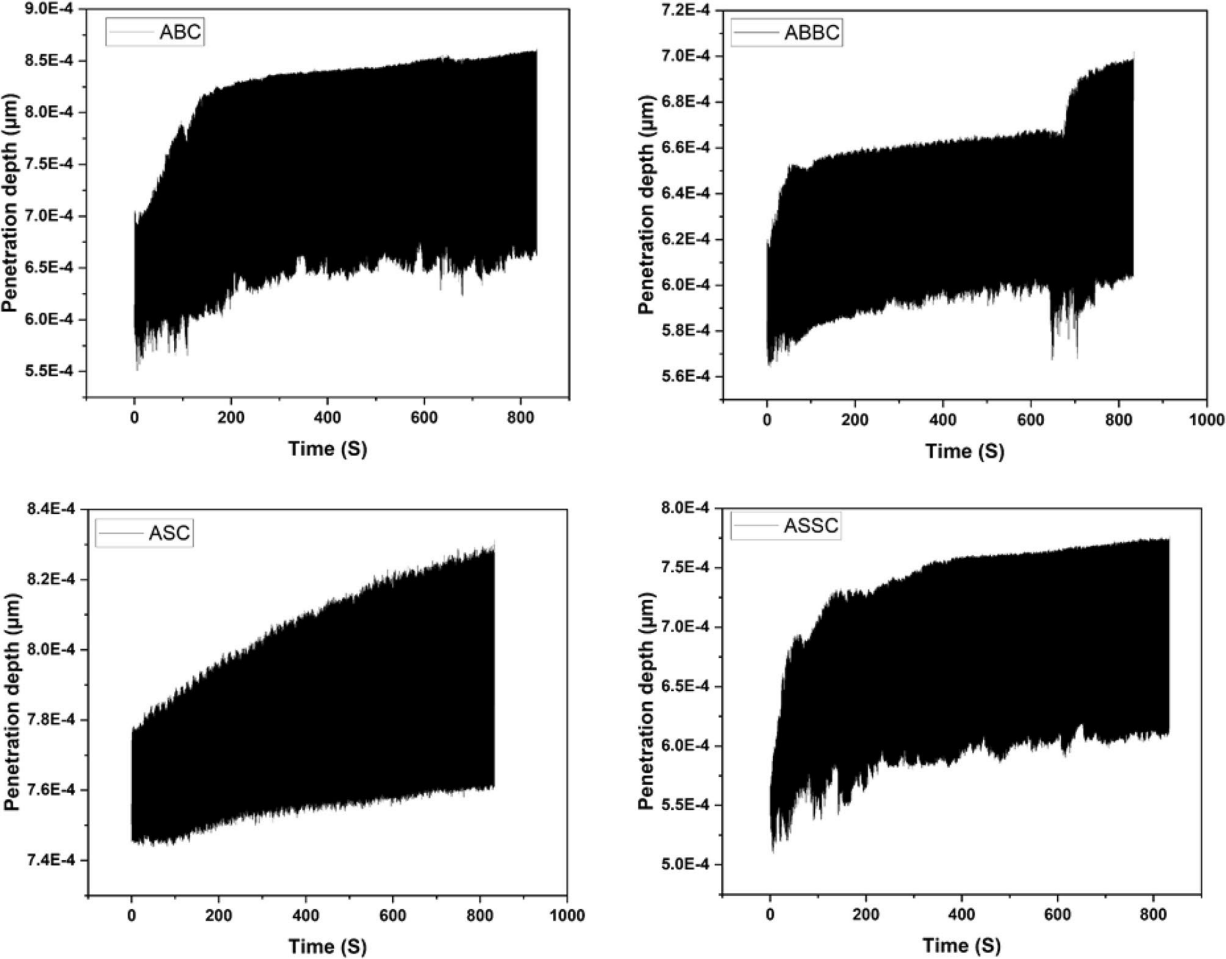
Scratch/penetration depth during tribo-testing with respect to time of the composite.

Figure 18 depicts how scratch depth varies with reinforcing. The inclusion of reinforcement lowers the penetration depth, as seen in both cases with B_4_C and SiC reinforcement. When compared to ABC, ABBC has a reduced scratch depth, while ASSC and ASC exhibit the same tendency as boron carbide reinforced composite. For ABBC, ABC, ASSC, and ASC, the penetration/scratch depths are 167.45 µm, 175.56 µm, 176.19 µm, and 187.24 µm, respectively. Figure 17 shows the minimum depth for ABBC composite. This occurs because more reinforcement improves the surface mechanical behavior of the aluminum nano composite, resulting in higher plastic deformation strength. Similarly, the highest width of scratch was observed for SiC reinforced composite and the least for B_4_C reinforced composite. Surface deformation is a component of tribological study, and the forces used during tribological testing cause the composite surface to deform in an elastic or plastic manner. Utilizing methods like surface profilometry, which measures the surface topography to assess the degree of surface deformation, surface deformation is frequently assessed. Figure 17 depicts the scratch width, which was 1624.88 µm for ASSC and 1077.94 µm for ABBC, respectively. The results revealed that the wear rate dropped dramatically with the more addition of SiC and B_4_C particles. Additionally, adding B_4_C particles resulted in less wear than adding SiC particles, indicating that B_4_C has a marginally higher wear resistance than SiC due to its higher hardness and stiffness. The enhanced behavior of the material allows SiC and B4C reinforcements to be added to aluminum matrix composites to strengthen their wear resistance. These composites' wear behavior can be influenced by the kind and quantity of reinforcement as well as the testing environment. The processing and testing conditions of these composites must be optimized in future research in order to improve wear performance in a variety of applications.

## Conclusion

Squeeze stir casting was used to create an aluminum nanocomposite with 8 and 12 vol% of B_4_C/SiC, and 2 vol% CNT. Tests for nanoindentation and nanoscratching were performed on the composite. To assess the micro-mechanical characteristics of the nanocomposite, indentations and scratches were applied to each sample. In addition, SEM, TEM, and surface profilometers were utilized to examine the samples' micrographs, intermetallic formation, and surface topography. The following conclusions has been drawn from present research work:

- Squeeze stir casting was used to create composites of (B_4_C + CNT)/Al and (SiC + CNT)/Al with 8, and 12 vol%. These composites' mechanical characteristics, microstructure development, and CNT absorptivity are all affected by the (B_4_C + CNT) composition.
- SEM micrographs show that the reinforcement in the composites, such as (B4C + CNT)/Al and (SiC + CNT)/Al, is homogeneous at various volumes of the reinforcement in addition to the presence of particle aggregation in a few locations of the microstructure.
- The presence (reinforcing phases) of B_4_C and SiC led to the presence of Al, B_4_C and Al_3_BC and Si, SiC and Al_4_C_3_ precipitates, respectively and it is confirmed through XRD peak and TEM EDS analysis. The density of unmolten particles and nano-precipitates in the composites as-fabricated increases when more reinforced components are added.
- The micro and nano hardness of the composites is influenced by the inclusion of both B_4_C and SiC and it is improved by addition of reinforcement. Increased hardness results from the presence of aluminum matrix and reinforced particles, which has low solidity. B_4_C offers greater resistance to deformation than SiC since It is an incredibly high solidity, low specific weight, high performance monolithic ceramic particle. When 12 vol% of B_4_C is added, 117 HV is measured as the maximum hardness. When 12 volume percent of SiC is added to an aluminum matrix, the maximum hardness is discovered to be 113 HV for Al/SiC composite. According to experimental findings, the composite’s hardness increased by 16% and 12%, respectively, as a result of the B_4_C and SiC reinforcement. The composite with the maximum hardness is ABBC.
- With increasing reinforcement content, the average micro and nano-hardness of the (B_4_C + CNT)/Al and (SiC + CNT)/Al composites rises, reaching a maximum micro hardness of 194 ± 2 HV_0.1_ and nano-hardness of 204.9 ± 5 GPa for the 12 vol% (B_4_C + CNT)/Al composites. The elastic modulus improved by 12% for 12 vol% of (B_4_C + CNT)/Al compared to (SiC + CNT)/Al. The improved properties are attributed to in-situ nano-precipitate production and a good coherent interface between the reinforcements and matrix. This study can serve as a guide for creating high-performance hybrid aluminum composites that are reinforced with (B_4_C + CNT) and (SiC + CNT).
- The topography of the scratched surface reveals the wear and friction behavior of the composite, and it is discovered that the wear rate drops from 3.8 × 10^–5^ mm^3^N^−1^ mm^−1^ to 2.7 × 10^–5^ mm^3^N^−1^ m^−1^, and the friction coefficient increases from 0.65 to 0.85, indicating a high that is 30% better than the corresponding limit.
- The topography analysis revealed the scratch depth and width and concluded that reinforcing reduces the depth and width. ASSC's composite scratch depth is 5% deeper than ABBC's. and ASSC's scratch width is 48% wider than that of ABBC composite.
